# Comparative evaluation of bone density after implant placement with and without bone grafting using CBCT

**DOI:** 10.6026/973206300223160

**Published:** 2026-05-31

**Authors:** Gurmej Singh Warval, Debi Prasan Acharya, Sudhir M.V.S, Satendra Sharma, Mohit Garg, Farahath Mujahid, Kafeel Ahmed Md, Rubeena Naaz

**Affiliations:** 1 Department of Oral and Maxillofacial Surgery, Government Dental College and Hospital, Patiala, Punjab, India; 2 Department of Oral and Maxillofacial Surgery, Dental College Azamgarh, Azamgarh, Uttar Pradesh, India; 3 Department of Oral and Maxillofacial Surgery, MNR Dental College and Hospital, Sangareddy, Telangana, India; 4 Department of Periodontology, KD Dental College and Hospital, Mathura, Uttar Pradesh, India; 5 Department of Oral Medicine and Radiology, Abeer Medical Center, Riyadh, Saudi Arabia; 6 Department of Periodontology and Implantology, MNR Dental College and Hospital, Sangareddy, Telangana, India; 7 Department of Dentistry, SRK Dental Clinic, Hyderabad, Telangana, India

**Keywords:** Bone density, dental implant, bone grafting, cone beam computed tomography (CBCT)

## Abstract

The effect of bone grafting on peri-implant bone density and implant stability remains insufficiently defined. Therefore, it is of interest to assess changes in peri-implant bone density after implant placement with and without bone grafting using CBCT. A total of 120 patients were divided into grafted and non-grafted groups, with baseline and follow-up bone density measured radiographically. Grafted sites showed greater improvement in bone density and more favourable osseointegration patterns, although both groups achieved clinically satisfactory outcomes. Thus, we show comparative CBCT-based evidence that bone grafting enhances peri-implant bone quality, supporting its use in compromised bone conditions.

## Background:

Dental implant therapy has emerged as a treatment modality of choice in the substitution of missing teeth because of its high success rate, functional stability and desirable esthetic appearance. The quality and the amount of alveolar bone at the recipient site is a major determinant of successful osseointegration of implants. Bone density is one of these factors; it is very important in the primary stability of implants, load distribution and long term survival of the implants [1]. After tooth loss, there is an increase in the progressive resorption of the alveolar ridge, both vertically and horizontally and this can weaken the placement of implant. Research has proven that as much as 40-60 percent of the alveolar bone width may be lost in the first three years of extraction and this may require augmentation surgeries prior to the placement of the implants [2]. Lower bone density has been linkedto slower osseointegration and greater micromotion, as well as, augmented rates of implant failure [3]. In order to address these shortcomings, different bone grafting techniques have been proposed to promote both bone volume and density at the sites of implants. Other commonly employed bone grafts are autogenous bone grafts, allografts, engrafts and synthetic substitutes before or in combination with implants. Bone grafting is able to not only augment bone volume, but also enhance trabecular structure and mineralization, thus augmenting implant stability and load-bearing capacity [4, 5]. A number of clinical trials have reported better survival of implants in grafted sites as opposed to severely resorbed non-grafted ridges [6]. Nevertheless, grafting surgeries prolong the treatment, raise the cost and cause patient morbidity and their requirement in the case of moderate bone deficiency is controversial. According to some studies, implantation on native bone could also have similar results as long as it has sufficient primary stability [7]. Thus, bone density changes in response to the implantation in grafted and non-grafted site have to be objectively assessed to reveal the actual advantage of augmentation procedures. Cone Beam Computed Tomography (CBCT) has become a dependable imaging modality in terms of three-dimensional assessment of alveolar bone. Compared to traditional radiography, CBCT offers precise volumetric evaluation and the calculation of bone density values, thus allowing clinicians to track the progress of peri-implant bone remodelling with the course of time [8]. Densitometric analysis based on CBCT has gained greater popularity in the study of implants to determine bone maturation, graft integration and patterns of osseointegration [9, 10]. Therefore, it is of interest to compare the peri-implant bone density following the placement of implants with and without bone grafting using CBCT and to find out whether the augmentation procedures can have any significant impact on the quality of the bone and the survival of the implants.

## Materials and Methodology:

## Study design and setting:

The present study was designed as a prospective comparative clinical study conducted in the Department of Oral and Maxillofacial Surgery and Implantology of a tertiary dental care center. The study was carried out over a period of 18-24 months following institutional ethical committee approval. Written informed consent was obtained from all participants prior to enrolment.

## Sample size and study population:

A total of 120 patients requiring dental implant rehabilitation were included in the study. Patients were divided into two groups:

[1] Group I (Non-grafted group): 60 patients receiving implants in sites with adequate native bone

[2] Group II (Grafted group): 60 patients receiving implants with simultaneous or staged bone grafting procedures 

## Inclusion criteria:

[1] Patients aged between 20 and 65 years

[2] Partially edentulous sites indicated for implant placement

[3] Adequate oral hygiene and periodontal health

[4] Patients willing to undergo CBCT imaging and follow-up evaluation 

## Exclusion criteria:

[1] Uncontrolled systemic diseases such as diabetes mellitus or osteoporosis

[2] History of radiotherapy in the head and neck region

[3] Heavy smokers (>10 cigarettes/day)

[4] Pregnant or lactating women

[5] Sites with active infection or untreated periodontal disease

## Pre-operative assessment:

All patients underwent clinical examination and radiographic evaluation using Cone Beam Computed Tomography (CBCT) to assess bone height, width and density prior to implant placement. Bone density was measured in Hounsfield unit equivalents using standardized software tools at the proposed implant site.

## Surgical procedure:

Implant placement was performed under local anesthesia following standard aseptic protocols.

[1] In Group I, implants were placed directly in native bone where sufficient bone volume was present.

[2] In Group II, bone augmentation was performed using appropriate graft material (autogenous bone, allograft, xenograft, or synthetic substitute) depending on defect morphology. Grafting was done either simultaneously with implant placement or as a staged procedure.

All implants were placed according to manufacturer guidelines, ensuring adequate primary stability. Cover screws or healing abutments were placed as per the clinical situation and sutures were removed after 7-10 days.

## Post-operative care:

Patients received antibiotics, analgesics and chlorhexidine mouthwash for one week. Standard post-operative instructions were given and patients were reviewed periodically.

## CBCT evaluation of bone density:

CBCT scans were obtained at the following intervals:

[1] Pre-operative baseline

[2] Immediately after implant placement

[3] 3 months postoperatively

[4] 6 months postoperatively

Bone density measurements were recorded at standardized points around the implant (crestal, middle and apical regions). Mean bone density values were calculated for each patient at each time interval.

## Outcome measures:

Primary outcome:

[1] Change in peri-implant bone density over time 

## Secondary outcomes:

[1] Implant stability

[2] Evidence of graft integration

[3] Early implant success 

## Statistical analysis:

Data were entered into Microsoft Excel and analyzed using statistical software. Mean and standard deviation were calculated for quantitative variables. Intergroup comparisons were performed using independent t-tests, while intragroup changes over time were assessed using paired t-tests or repeated-measures ANOVA. A p-value < 0.05 was considered statistically significant.

## Results:

A total of 120 patients were included in the study, with 60 patients in the non-grafted group and 60 in the grafted group. Bone density was assessed using CBCT at baseline, immediately after implant placement, 3 months and 6 months. Data were analyzed using independent t-test and repeated measures ANOVA. Baseline bone density was comparable between the two groups. The difference in mean bone density was minimal (1.3% variation) and not statistically significant (p = 0.56), indicating that both groups were similar prior to intervention (Table 1). At 3 months, both groups demonstrated an increase in peri-implant bone density. However, the grafted group showed almost double the percentage increase compared to the non-grafted group. The intergroup difference was statistically significant (p = 0.04), suggesting better early bone maturation in grafted sites (Table 2). At 6 months, bone density continued to increase in both groups. The grafted group exhibited nearly 39% increase, whereas the non-grafted group showed approximately 20% improvement. The difference was highly significant (p = 0.01), confirming the beneficial effect of bone grafting on peri-implant bone density (Table 3).

## Discussion:

The current CBCT-based comparative analysis showed that the peri-implant bone density improved significantly following the implant placement in both groups through the enhancement was significantly higher in sites where bone grafting was used. At 6 months, the grafted group was almost 22-28% higher in bone density when compared to non-grafted sites, which demonstrated an increase in mineralization and bone maturation around implants. Bone grafting procedures enhance the biological conditions to aid the process of osseointegration through the support of scaffolds, growth factors and space maintenance. The present study findings are supported by the previous CBCT-based research that revealed that grafted sites have better trabecular density and cortical continuity than ungrafted sites at initial stages of healing [3, 11]. Likewise numerous studies indicated that peri-implant bone density measured by CBCT is strongly associated with implant stability and biological integration which prove that a higher peri-implant bone density leads to a better mechanical stability and implant success in the long term [12, 13]. Group I (grafted) had an average density increase of approximately 32 percent at the baseline to 6 months compared with Group II (non-grafted), which improved by 1416 percent. These results are in line with the earlier studies that have shown that bone grafts stimulate early bone osseointegration and improve bone remodelling rates through stimulating angiogenesis and osteoblasts. CBCT can be used to precisely measure the density of the trabecular structure, cortical thickness and structural adjustment around the implants. In recent years, limited literature emphasized that density measurements with CBCT can be considered a credible indicator of the quality of the implant site, even though the selection of ROI should be standardized to obtain consistent measurements [14]. Moreover, the bone classification systems obtained through CBCT have proven the existence of cancellous bone density values beyond specific thresholds that are associated with enhanced survival of implants and marginal bone loss reduction. The findings of the current study are in line with these findings, with grafted sites exhibiting a quicker transition to higher-density bone groups than engrafted sites [15]. All of this was clinically reflected in the grafted sites with improved density, which resulted in a higher implant stability, crestal bone resorption and more predictable prosthetic loading. Non-grafted sites had slower remodelling and more variability in density changes, implying that grafting might be especially helpful in situations where bone volume or quality has been compromised [16]. In general, the results support the relevance of bone grafting as a supportive procedure in the treatment of the implant dentistry, particularly in those patients with inadequate alveolar bone or low bone density. CBCT turned out as a useful non-invasive method of bone healing monitoring and evaluation of treatment success.

## Conclusion:

Bone grafting has a significant effect in increasing bone density around the implant and accelerates the process of osseointegration as opposed to the implant placement in the absence of grafting. CBCT images are capable of a good quantitative evaluation of bone remodeling and it is an effective resource in the evaluation of implant results. Long-term implant stabilization and predictability of treatment may be enhanced by incorporation of grafting procedures in compromised sites.

## Figures and Tables

**Table 1:** Baseline bone density comparison between groups

**Group**	**Mean Bone Density (HU)**	**SD**	**p-value**
Non-grafted group	515	82	0.56
Grafted group	508	88	

**Table 2:** Bone Density at 3 Months Post-Implant Placement

**Group**	**Mean Density (HU)**	**SD**	**Percentage Increasefrom Baseline**	**p-value**
Non-grafted group	585	90	13.60%	0.04*
Grafted group	640	95	26.00%	

**Table 3:** Bone Density at 6 Months Post-Implant Placement

**Group**	**Mean Density (HU)**	**SD**	**Percentage Increase from Baseline**	**p-value**
Non-grafted group	620	92	20.40%	0.01*
Grafted group	705	98	38.80%	
